# Machine‐Learning Microfluidic Minute‐Scale Microorganism Metrics Monitoring(M6)

**DOI:** 10.1002/advs.202521106

**Published:** 2026-04-15

**Authors:** Ning Yang, Jiahao Ding, Si Chen, Lijie Yan, Shichao Ding, Lavonda Li, Junyi Sun, Haodong Liu, Tongge Li, Ning Liu, Mingji Wei, Xiaoyong Zhu, Xiaobo Zou, Shouqi Yuan, Xingcai Zhang

**Affiliations:** ^1^ School of Electrical and Information Engineering Jiangsu University Zhenjiang China; ^2^ World Tea Organization Cambridge Massachusetts USA; ^3^ Fluid Machinery Center Jiangsu University Zhenjiang China; ^4^ School of Medicine Stanford University Stanford California USA; ^5^ Fuwai Central China Cardiovascular Hospital Central China Fuwai Hospital of Zhengzhou University Zhengzhou China; ^6^ Department of Chemical and Nano Engineering University of California San Diego, La Jolla California USA; ^7^ Department of Materials Science and Engineering Stanford University Stanford California USA; ^8^ School of Food and Biological Engineering Jiangsu University Zhenjiang China

**Keywords:** epidemic early warning, global public health, machine learning microfluidics, microorganism aerosol detection, minute‐scale monitoring

## Abstract

On‐site monitoring of microorganisms remains challenging because of low concentrations, strong background interference, and dynamic aerosol diffusion, particularly for aerosol‐transmitted pathogens. Here, we report a rapid detection platform that integrates a Puri‐focusing microfluidic chip, electrochemical impedance spectroscopy (EIS), and machine learning for the analysis of airborne microorganisms. Guided by fluid‐dynamic design and laminar‐flow focusing, the chip achieved a 95.8% separation efficiency for 5 µm target particles. African swine fever virus (ASFV) was used as a model pathogen. Impedance features, including modulus, real and imaginary components, and phase angle, were extracted from aerosol samples and analyzed using multiple machine learning classifiers. Five‐fold cross‐validation identified Random Forest (RF) as the optimal model, achieving 95.2% classification accuracy. The platform reached a system‐level detection limit of 188 TCID50/mL for air‐sampled aerosols and showed high concordance with enzyme‐linked immunosorbent assay (ELISA) results. Each detection cycle required less than 1 minute. This integrated strategy offers a feasible route for rapid on‐site monitoring of aerosol‐transmitted microorganisms in public health, agriculture, livestock farming, and production safety.

## Abstract 2

A Puri‐focusing microfluidic chip integrated with electrochemical impedance spectroscopy (EIS) and machine learning enables minute‐scale on‐site detection of African fwine fever virus (ASFV) aerosols. The system achieves 95.8% particle separation and 95.2% classification accuracy, supporting rapid monitoring of airborne pathogens in complex environments.

## 1 Introduction

Microorganisms are ubiquitous in nature and profoundly impact human activities [1, 2, 3, 4]. Pathogenic microorganisms, such as drug‐resistant bacteria and viruses, threaten global public health, livestock farming, and production safety [5, 6, 7, 8, 9]. Studies on large‐scale epidemics—including the global outbreaks of COVID‐19, Influenza, and African swine fever—have demonstrated that viruses can be transmitted across regions in aerosol form, causing respiratory tract infections in both humans and animals [10, 11, 12]. However, it is very difficult to effectively collect these low‐concentration microparticles. Furthermore, due to the complexity of the environmental matrix, conventional detection methods rely on cumbersome sample processing and high‐precision equipment, making it difficult to monitor the dynamic variations of pathogens in real time [13, 14]. Therefore, establishing a real‐time, highly sensitive, integrated sampling‐detection method is essential for the effective prevention and control of pathogenic microbial aerosols.

Microbial aerosol collection technologies primarily rely on physical separation principles, including sedimentation, filtration, impaction, and adsorption [15, 16]. While these methods can effectively collect aerosol particles from the air, they typically require large‐volume equipment and often alter the biological properties of the particles during the sampling process. Furthermore, their limited anti‐interference capability restricts their application in on‐site aerosol detection requiring high precision [17]. Microfluidic technology, owing to its ability to precisely manipulate fluids at the microscale, offers a novel solution for the efficient separation and enrichment of aerosol particles [18, 19]. By designing specialized microfluidic chip structures, it is also possible to purify microparticles of specific sizes, thereby enhancing sample concentration. Consequently, constructing a detection platform based on a microfluidic‐sensing synergy architecture can enable dimension‐reduced detection of complex components, thereby improving the limit of detection(LOD) and accuracy.

Existing microbial detection methods, including molecular or immunological techniques such as PCR, ELISA, and CRISPR, offer high specificity and accuracy. However, these approaches are heavily equipment‐dependent, involve cumbersome sample processing, and suffer from detection lag [20, 21, 22, 23, 24]. While some rapid detection technologies, such as light scattering and biofluorescence sensing, offer a degree of real‐time capability, they are susceptible to interference from particle morphology and complex matrices [25, 26]. Conversely, Electrochemical Impedance Spectroscopy (EIS) provides an ideal label‐free, real‐time platform [27]. By immobilizing recognition molecules on the electrode surface, EIS can instantaneously report changes in impedance upon particle binding events, generating multi‐frequency domain feature signals [28, 29, 30, 31]. Nevertheless, EIS is prone to interference from non‐specific adsorption on the electrode surface and complex matrices. The resulting multi‐frequency impedance signals collected from microorganisms often exhibit high overlap and non‐linear characteristics, making accurate classification based solely on a fixed threshold challenging.

In recent years, machine learning algorithms have been integrated into the impedance signal analysis process, transfor2g the EIS system from a simple ‘physical sensor’ into an ‘intelligent classifier’ with self‐learning and discriminatory capabilities [32]. By training models on multi‐frequency domain impedance fingerprints, this approach enables dimension‐reduced detection and intelligent discrimination of complex information, thereby effectively distinguishing between pathogens and non‐target interferents [33]. Consequently, machine learning can effectively compensate for the limitations of EIS in the on‐site rapid detection of microbial aerosols. Furthermore, it automates the data analysis process, which can satisfy the demand for immediate high‐throughput detection of different categories of microbial aerosols.

Therefore, we propose an automated detection platform that integrates a ‘Puri‐focusing’ microfluidic structure with EIS detection, achieving high‐efficiency enrichment and label‐free, real‐time identification of target microbial particles. In this study, we utilized an aerosol generator to prepare microbial aerosol samples and established an aerosol chamber to simulate particle propagation in the air. Using pathogenic microbial aerosols as an example, we designed a background filtration zone and a curved vortex structure to achieve an enrichment efficiency of 95.8% for particles with a targeted diameter of 5 µm. We employed EIS to analyze the enriched samples, selecting 10 Hz as the characteristic detection frequency. Furthermore, we compared multiple representative machine learning algorithms for classification analysis and ultimately selected the RF algorithm as our model, which achieved a classification accuracy of 95.2%. The experiments detailed in this paper were conducted using ASFV aerosol, and the method's accuracy was validated against the widely commercialized ELISA. The results demonstrated high concordance, and the entire detection process was controlled to be completed within one minute. Compared to many existing detection methods used for large‐scale epidemic transmission, our study establishes an automated “sampling–purification–detection–identification” workflow and methodology based on experimental results obtained under laboratory conditions emulating on‐site environments, providing a scalable technological pathway for rapid on‐site detection of pathogenic aerosols.

## 2 Methods and Materials

### 2.1 Materials and Sample Preparation

**FIGURE 1:**
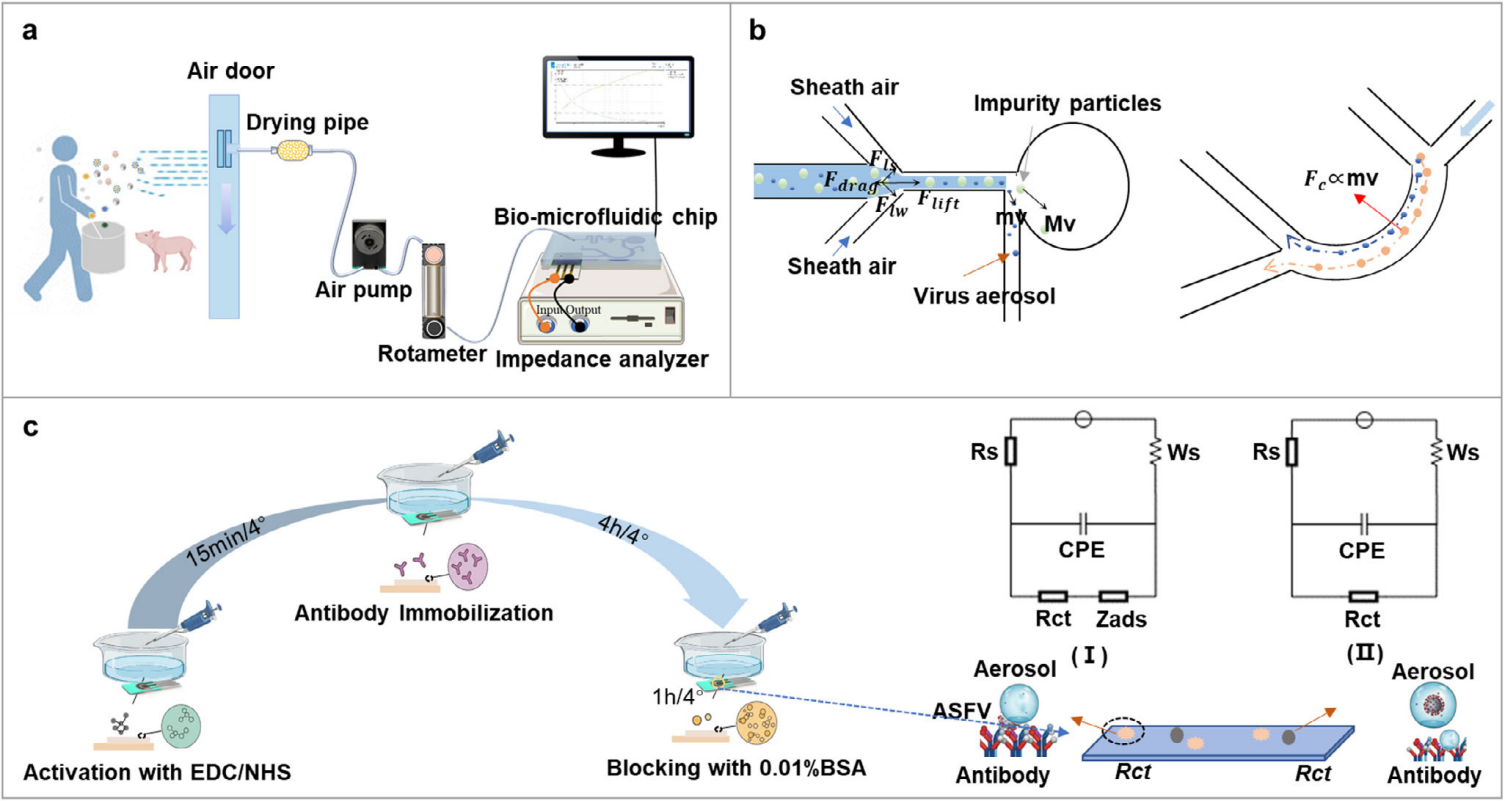
Microfluidic chip combined with electrochemical impedance spectroscopy for detection of African swine fever virus aerosols. (a) Experimental platform including the aerosol chamber, microfluidic chip, and impedance analyzer. (b) Schematic diagram of particle flow in the microfluidic chip and the purification principle. (c) Preparation process of the biosensor detection chip and equivalent circuit detection models for different aerosol particles.

In this study, A Screen‐Printed Electrode (SPE) was employed as the sensing unit in the microbial aerosol detection platform (Figure S1), integrating a microfluidic chip with the SPE (Figure 1a). The eukaryotic monoclonal antibody against ASFV P30 and its corresponding antigen were purchased from Shanghai Yuduo Biotechnology Co., Ltd., and the ASFV, CSFV, and PRV virus solutions were provided by COFCO Corporation. Among them, ASFV is a large enveloped double‑stranded DNA virus, classical swine fever virus (CSFV) is an enveloped single‑stranded RNA virus, and pseudorabies virus (PRV) is an enveloped double‑stranded DNA herpesvirus. To ensure experimental safety, all virus samples were inactivated and used as biosafety‑compliant positive controls; the relative titers of the three virus solutions were TCID_50_ values of 10^7^,10^8^ and 10^8^, respectively. Furthermore, the equivalent TCID_50_ unit for viral concentration adopted in this study was derived from the pre‐inactivated titer data provided by the supplier. Accordingly, this unit is presented as a calibrated quantitative metric for aerosolized viral particles in the gas phase. The virus samples were stored at −20 °C according to the supplier's recommendations and were dissolved in a 37 °C water bath prior to use. In the experiments, ASFV aerosols served as the target detection virus, whereas the CSFV, PRV, and PBS aerosol groups were used as negative controls.

Previous studies have demonstrated that aerosol particles within the 2–5 µm range are more likely to carry viruses [34]. Although particle sizes in real‐world aerosols exhibit a continuous and heterogeneous distribution, discrete particle diameters are commonly adopted in controlled studies to represent characteristic size regimes. In this work, 3 µm, 5 µm, and 10 µm particles were selected to respectively represent smaller respirable particles, virus‐associated aerosol particles reported in prior studies, and larger background impurities. This size selection strategy allows systematic evaluation of separation and detection performance while retaining relevance to realistic aerosol conditions. The preparation of virus aerosols was as follows: 100 µL of ASFV solution was taken, 1 mL of 0.01 M PBS was added, and the mixture was ultrasonicated for 2 min to ensure uniform dispersion of particles. The preparation procedures for the CSFV and PRV samples were the same.

### 2.2 Construction of the Detection Platform

In the experiments, an aerosol generator was used to atomize the virus solution and PBS buffer to produce aerosol particles, and a blower was started to allow the aerosols to fully diffuse within the chamber, simulating the actual airborne transmission and collection process (Figure 1a). Subsequently, the flow at the inlet of the microfluidic chip was controlled via a flowmeter, and the sheath‑flow channels were adjusted to realize laminar focusing and preliminary purification of the target particle size. To establish the required electrolyte environment for detection and ensure the sensitivity and stability of subsequent measurements, PBS buffer solution was added to the electrode detection area.

Before the impedance‑detection module, the Zurich impedance analyzer was calibrated for accuracy to ensure the correctness of the output data. After microfluidic separation, the aerosol samples entered the detection region, and the analyzer collected electrochemical impedance signal data from the SPE electrode. The sampling frequency of the instrument was set to 13.39 k Sa/s, the working current frequency was set to 10 Hz, and the sampling voltage was 300 mV. During the sampling process, real‑time data were synchronously displayed on the host‑computer interface. All experiments were conducted under controlled laboratory conditions. For experimental convenience and to avoid additional system complexity at this stage, the detection setup was operated without dedicated electromagnetic shielding, i.e., in an electrically exposed configuration. Under these conditions, external signal interference was minimized through standardized experimental procedures rather than hardware‐level shielding. From an engineering perspective, this configuration is functionally equivalent to practical deployment scenarios in which electromagnetic interference is mitigated by conventional measures such as shielded enclosures or integrated system housings.

Although the experiments were performed in a laboratory environment, the detection system was evaluated under non‐ideal conditions by intentionally introducing particle impurities and non‐target interferents. This design was intended to partially emulate the complexity of realistic aerosol backgrounds and to assess the robustness of the integrated microfluidic–electrochemical impedance–machine learning framework. The stable discrimination performance observed under these conditions indicates a certain tolerance of the proposed system to background interference, supporting its potential applicability in more complex operating environments.

### 2.3 Microfluidic Chip Structure Design

**FIGURE 2:**
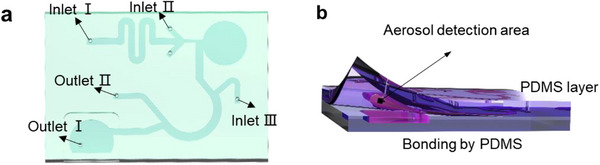
Schematic diagram of the microfluidic chip. (a) Arrangement of channel inlets and outlets. (b) Cross‐sectional view of the double‐layer structure, The microfluidic chip was fabricated by soft lithography, PDMS is used as the substrate material for both layers.

This study designed a microfluidic chip based on arc‑shaped curved channels and sheath‑flow focusing to achieve efficient separation and enrichment of microbial aerosols (Figure 2). The design is grounded in inertial separation and laminar focusing, maintaining a compact footprint while improving the accuracy and stability with which target particles enter the detection region. The chip incorporates a serpentine buffer region to fully disperse viral aggregates. Downstream, a 45° double‐sheath configuration is adopted in the main channel; this configuration substantially narrows the sample core width and increases the flow velocity, thereby enhancing the focusing effect. By controlling the width ratio and the flow‐rate ratio between the main channel and the sheath streams, precise focusing of target particles at the channel center can be achieved. The focused sample‐stream width *W_f_* is given by the following equation:

(1) $$W_{f}=W_{Sa}\cdot \frac{W_{Sa}\cdot v_{Sa}}{W_{Ss}\cdot v_{Ss}}$$

In the formula, *W_Sa_* represents the width of the sample injection channel, *v_Sa_* represents the sample injection speed, *W_Ss_* represents the width of the sheath flow channel, and *v_Ss_* represents the sheath flow velocity. Meanwhile, the flow velocity at the exit can be expressed as:

(2) $$v_{out}=\eta \cdot \frac{W_{Sa}}{W_{out}}\left(1+\frac{W_{Ss}\cdot v_{Ss}}{W_{Sa}\cdot v_{Sa}}\right)$$

where η represents the channel viscosity coefficient.

To enhance particle separation capability, the chip employs a curved‐channel structure. When the fluid flows through the microchannel, the particles deviate from the main path under the action of centrifugal force, which can be expressed as:

(3) $$F_{c}=\rho_{p}V_{p}\frac{v^{2}}{r}$$

In the formula, *F_c_* represents the centrifugal force experienced by the particles at the bend,ρ_*p*_ *V_p_* represents the particle mass, *v* represents the channel flow velocity, and *r* represents the radius of curvature. According to Dean theory, the secondary Dean flow intensity in the bend (De) is expressed as [35]:

(4) $$D_{e}=\sqrt{R_{e}\cdot \frac{R_{h}}{r}},R_{h}=\frac{WH}{W+H}$$

In the formula, *R_e_* represents the Reynolds number, *R_h_* represents the hydraulic radius, *W* represents the channel width, and *H* represents the channel height. Accordingly, by adjusting the aspect ratio of the channel (*W/H*) and the radius of curvature *r*, the Dean flow field structure can be effectively regulated, thereby optimizing the enrichment efficiency and spatial distribution of particles. According to Stokes’ law, particles in the channel are acted upon jointly by the inertial lift *F_lift_* ​, the viscous drag *F_drag_*, and the transverse force induced by Dean vortices *F_Dean_* (Figure 1b). Under steady‐state separation conditions these forces satisfy:

(5) $$F_{lift}+F_{drag}+F_{Dean}=0$$

Based on the above principles, the proposed structure can, by tuning physical parameters, achieve effective purification of target viral aerosol particles within the detection region, thereby providing a reliable input for subsequent impedance measurements.

### 2.4 Immuno‐Functionalization of the Electrode

Prior to the experiment, to enhance the efficiency of covalent protein coupling, the electrode surface underwent a chemical activation treatment (Figure 1c). The specific procedure was as follows: first, the SPE electrode was immersed in an activation solution containing EDC and NHS and incubated for 15 min at 4°C to activate the carboxyl groups on the electrode surface. Subsequently, it was rinsed three times with 10 mM PBS buffer (pH 7.4) to thoroughly remove any unreacted EDC/NHS residues. For the antibody immobilization stage, 10 µL of a 0.5 mg/mL P30 ASFV monoclonal antibody solution was drop‐casted onto the activated electrode surface and incubated for 4 h at 4°C, thereby immobilizing the antibody on the electrode via covalent coupling between the antibody's surface amine groups and the activated carboxyl groups. Following this, the modified electrode was dried at room temperature (25°C) for 30 min and washed thoroughly with PBS buffer to remove unbound antibody molecules. To block non‐specific binding sites on the electrode surface, a 0.01 mol/L BSA solution was added and incubated at 4°C for 1 h to complete the blocking treatment. Finally, the electrode was dried at 37°C for 30 min to enhance the stability of the BSA blocking layer, thereby improving the anti‐interference capability and signal‐to‐noise ratio for subsequent detections.

### 2.5 Principle of EIS Detection and Experimental Parameters

In this paper, EIS was employed for the qualitative and quantitative analysis of virus aerosols. EIS is a non‐destructive analytical technique that probes charge transfer behavior at electrode interfaces by applying a small‐amplitude AC signal and measuring the system's impedance response across a range of frequencies. When the electrode surface is functionalized with ASFV‐specific antibodies, the binding of virus particles alters the electrochemical properties of the electrode‐electrolyte interface. This, in turn, affects the parameters of the corresponding equivalent circuit model and produces discernible features in the impedance spectrum (Figure 2). To interpret the impedance changes associated with the specific binding of the target virus, two equivalent circuit models were developed. Specifically, in the absence of a binding event—either because the target virus is not present or is otherwise inaccessible within the aerosol particle—the system is represented by the virus‐free model (I). The equivalent circuit for this model is defined as:

(6) $$Z_{no-Virus}=R_{s}+\frac{R_{ct}}{1+R_{ct}Z_{CPE}}+Z_{w}$$

Conversely, when ASFV on the aerosol surface engages in specific binding with the antibodies on the electrode, the system corresponds to a virus‐detection state, which is described by Model (II), with the following equivalent circuit:

(7) $$Z_{Virus}=R_{s}+\frac{1}{Z_{CPE}+\frac{1}{R_{ct}+Z_{ads}}}+Z_{w},Z_{w}=R_{d}\frac{\tanh\sqrt{j\omega \tau }}{\sqrt{j\omega \tau }}$$

where: *R_s_* is the solution resistance, *Z_CPE_* is the non‐ideal double‐layer capacitance at the solution‐electrode interface (modeled as a Constant Phase Element), *R_ct_* is the charge transfer resistance, *Z_ads_* is the additional impedance introduced by virus binding, and *Z_w_* represents the finite‐layer diffusion impedance (Warburg element). As the model indicates, in the low‐frequency region, *Z_Virus_* ≈ R_*s*_+R_*ct*_+Z_*w*_, while in the high‐frequency region, the impedance behavior is primarily influenced by *Z_ads_*.

EIS measurements were conducted using a Zurich Instruments Impedance Analyzer. For all experiments, an AC excitation voltage of 300 mV was applied, and the sampling frequency was set to 10 Hz. The impedance response was scanned over a frequency range of 5 Hz to 1 MHz. This range was selected because the characteristic impedance differences between various aerosol samples were most pronounced in the low‐frequency region (5 Hz–1 kHz), while the high‐frequency data was used to assess the overall system impedance.

The EIS detection unit was directly coupled to the outlet of the microfluidic separation chip, allowing for the immediate measurement of aerosolized particles within the conductive PBS environment. To ensure data quality and minimize noise, the system was thoroughly flushed with PBS buffer before each experimental run to establish consistent initial conditions. A comprehensive dataset was collected, consisting of 70 sets of impedance data for each of the four sample categories (ASFV, CSFV, PRV, and a mixed group), totaling 280 data sets. Each data set included key parameters such as impedance modulus, the real and imaginary components of impedance, and phase angle.

To obtain robust impedance spectra for curve analysis and characteristic‐frequency selection, a median‐based averaging method was applied to the impedance spectra acquired by the impedance analyzer. This processing suppresses transient fluctuations and sporadic spikes in repeated measurements, while preserving the intrinsic spectral characteristics (S1).

### 2.6 Classification Model Construction

Machine learning has greatly advanced science especially biomedicine [36, 37, 38, 39, 40, 41, 42, 43, 44, 45, 46, 47, 48, 49, 50, 51, 52]. To automate the classification of virus aerosol samples from the electrochemical impedance data, several machine learning algorithms were employed. The parameters collected by the impedance analyzer, including the impedance magnitude (|Z|), phase angle, and the real and imaginary components, were selected at the characteristic frequency of 10 Hz from the impedance spectra and used as input features for machine‐learning–based classification. This feature‐selection strategy was adopted to balance classification effectiveness with computational simplicity. The full impedance spectra were first used for spectral analysis and characteristic‐frequency identification, and 10 Hz was selected because this low‐frequency region showed the largest inter‐class impedance separation together with the narrowest confidence interval across repeated measurements. At this frequency, the electrochemical response is primarily governed by interfacial charge‐transfer and adsorption‐related processes, which are most sensitive to the specific virus–antibody interactions on the functionalized electrode surface. Accordingly, the impedance magnitude, phase angle, and the real and imaginary components at 10 Hz were retained as a compact four‐dimensional feature set. This simplified representation reduces feature redundancy and facilitates efficient classification, while preserving the principal electrochemical information most relevant to target recognition. Prior to model training, all selected impedance features were normalized using z‐score normalization to eliminate scale differences among impedance parameters. The full impedance spectra were used for spectral analysis and characteristic‐frequency selection, rather than being directly used as multi‐frequency inputs to the machine‐learning models. While four categories of virus‐related samples (ASFV, CSFV, PRV, and mixed aerosols) were prepared in the experimental stage, the machine learning task in this study was intentionally formulated as a three‐class classification problem, with labels assigned as negative, positive, and mixed. This design was adopted because the objective of the current platform is not to develop a comprehensive pathogen‐oriented multi‐class classifier, but to assess whether the integrated sensing system, under the present configuration, can reliably discriminate target‐positive aerosols, target‐negative control aerosols, and mixed or interfering aerosol conditions. For model development and validation, the total dataset was randomly partitioned into a training set (70%) and a testing set (30%). A five‐fold cross‐validation scheme was implemented on the training data to rigorously evaluate the generalization performance of each model. We screened and compared algorithm categories, including ensemble methods, decision trees, support vector machines (SVMs), clustering, and Bayesian algorithms, drawn from various types of machine learning algorithms (Table S1). We compared five commonly used classifiers—Support Vector Machine (SVM), Random Forest (RF), K‐Nearest Neighbors (KNN), Gaussian Naive Bayes and Gradient Boosting to identify the most effective algorithm for this task (Table 1). The optimal model was selected based on a comprehensive evaluation of its performance metrics, including accuracy, precision, recall, and F1‐score. Given the limited dataset size and the compact four‐dimensional feature space used in this study, we did not further pursue more complex algorithms, because under such conditions models with higher complexity may be more prone to overfitting and may not necessarily yield a meaningful improvement in classification accuracy or generalization performance.

**TABLE 1:** Categories and Characteristics of the Selected Algorithm.

	Decision Tree Algorithm	Ensemble Algorithm	Clustering Algorithm	Bayesian Algorithm	SVM Algorithm
**Algorithm Types**	ID3、C4.5、CART、RF	Bagging、Boosting	KNN、DBSCAN、EM	Gaussian‐NB、NB、VNB	L‐SVM、M‐SVM、K‐SVM、S‐SVM
**Algorithm Features**	Intuitive rules and easy to interpret	Sequential learning, high accuracy; Strong resistance to overfitting	Unsupervised learning, focuses on internal data structure	Probability‐based, computationally efficient	Proficient at hand‐ling nonlinearity and small sample data

## 3 Result and Discussion

### 3.1 Microfluidic Chip Simulation and Experimental Results

**FIGURE 3:**
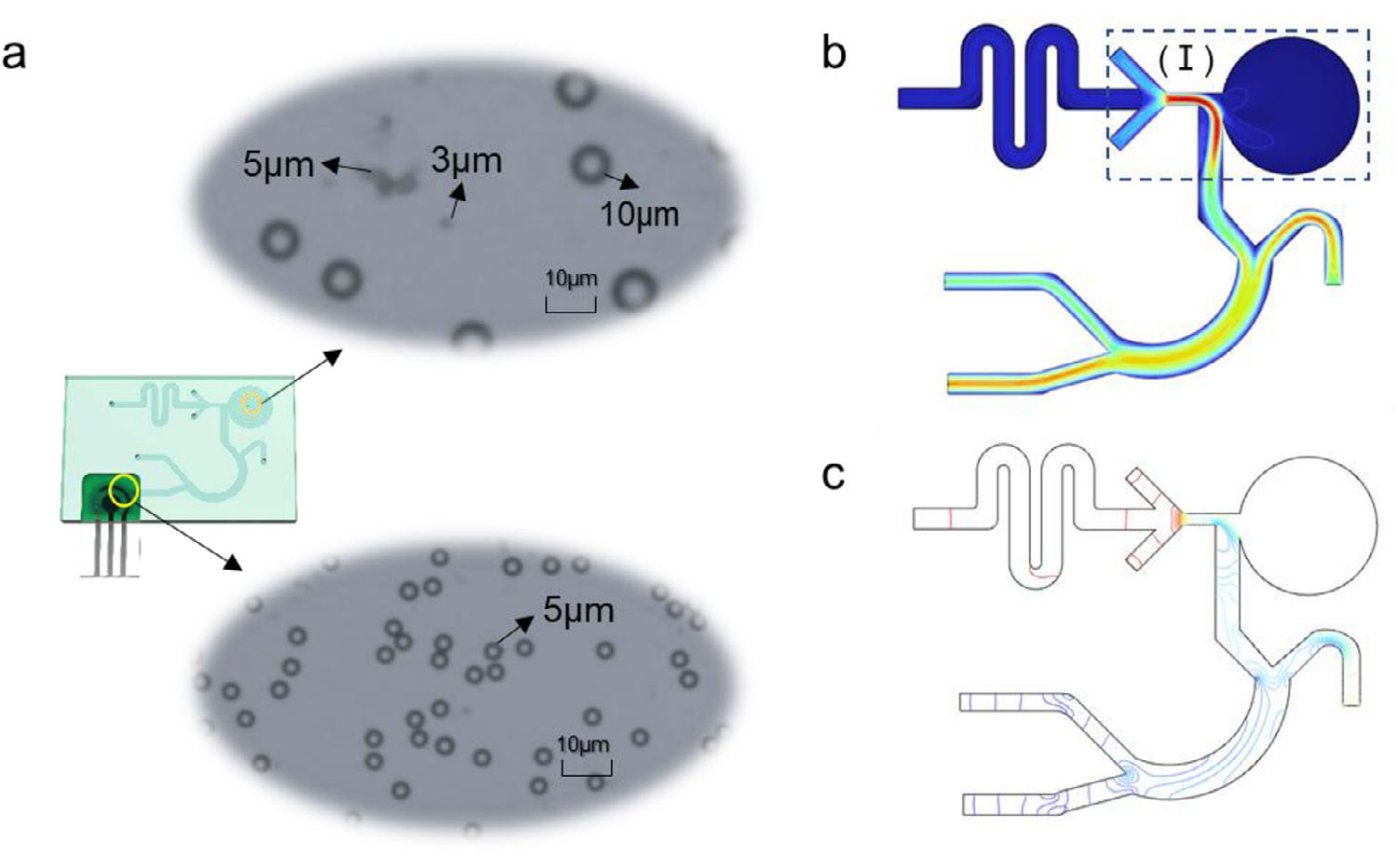
Microfluidic chip structure and COMSOL simulation results. (a) Experimental results of the impurity separation zone and the target particle enrichment zone. (b) Flow rate distribution map of the fluid within the chip when Inlet I is 12.5 mL/min, Inlet II is 50 mL/min, and Inlet III is 120 mL/min. (c) Pressure contour plot distribution within the microfluidic chip.

To achieve efficient collection and signal enhancement of virus aerosol particles from the air, a microfluidic chip integrating buffering, separation, and focusing functions was designed in this study (Figure 3a). The chip structure comprises three functional zones: a serpentine buffer zone to break up aggregated aerosol particles and improve particle uniformity before separation; a right‐angle separation zone for the initial removal of large‐diameter impurity particles to reduce background interference; and a y‐shaped separation structure that uses a dual sheath flow to compress the particle stream to the channel's center, leveraging centrifugal forces and Dean vortices in the curved channel to achieve secondary separation and enrichment of different‐sized particles. By introducing sheath flows at Inlet II and Inlet III respectively (Figure 3b), the design not only guides particles along the channel centerline to improve separation precision but also reduces the chip's footprint, enhancing its overall compactness (Figure S2).

The chip structure was simulated and optimized using COMSOL Multiphysics software. A physical field model incorporating both laminar flow and particle tracing modules was established to investigate the dynamic trajectories of different‐sized particles under various combinations of sheath and sample flow rates. Within the model, 5 µm particles were designated as the target particles, those larger than 5 µm were set as impurity particles, and particles smaller than 5 µm were considered secondary candidate particles for detection. To run the simulation, 100 particles of each size category were randomly released.

The simulation results indicated that in the curved channel region, the centerline flow velocity is higher than at the sides, causing the fluid to follow the outer curve and form Dean vortices (Figure 3b). An analysis of the laminar velocity and pressure fields, combined with the time‐domain particle tracing results, revealed the migration patterns of particles within the chip. In the first circular collection chamber, large particles deviate from the main stream due to inertial effects and enter the collection zone. Here, they are subjected to greater frictional drag, which prevents them from easily returning to the main channel, thus achieving the initial separation of large‐diameter impurities; smaller particles, however, continue along the main channel. In the downstream curved channel, the Dean secondary flow exerts differential lateral forces on particles of varying sizes, causing the larger particles to migrate further toward the outer wall, which completes the secondary separation and enhances the overall separation efficiency.

Based on the particle tracing results, we performed a numerical analysis of the particle trajectories and quantities within the chip to better adjust the chip's parameters and improve its performance. We calculated the number of particles at Outlet 1 and Outlet 2, respectively, as well as the number of particles retained within the chip. According to an analysis of the data provided by the software, the collection efficiency, separation efficiency, and retention loss rate of the target particles were calculated. The formulas for these calculations are as follows:

(8) $$\begin{array}{c} \eta_{c}=\left(\frac{A_{i}}{P_{i}}\right)\times 100\% \end{array}$$

(9) $$\eta_{s}=\left(\frac{N_{i}}{P_{o}}\right)\times 100\%$$

(10) $$\eta_{loss}=1-\frac{\left(N_{1}+N_{2}\right)}{P_{i}}\times 100\%$$

where *A_i_* is the number of particles with diameter *i* at outlet, *N_i_* is the total number of particles at outlet *i, P_i_* is the total number of particles with diameter *i* entering the channel, and *P_o_* is the total sum of particles of all diameters collected at the outlet.

**FIGURE 4:**
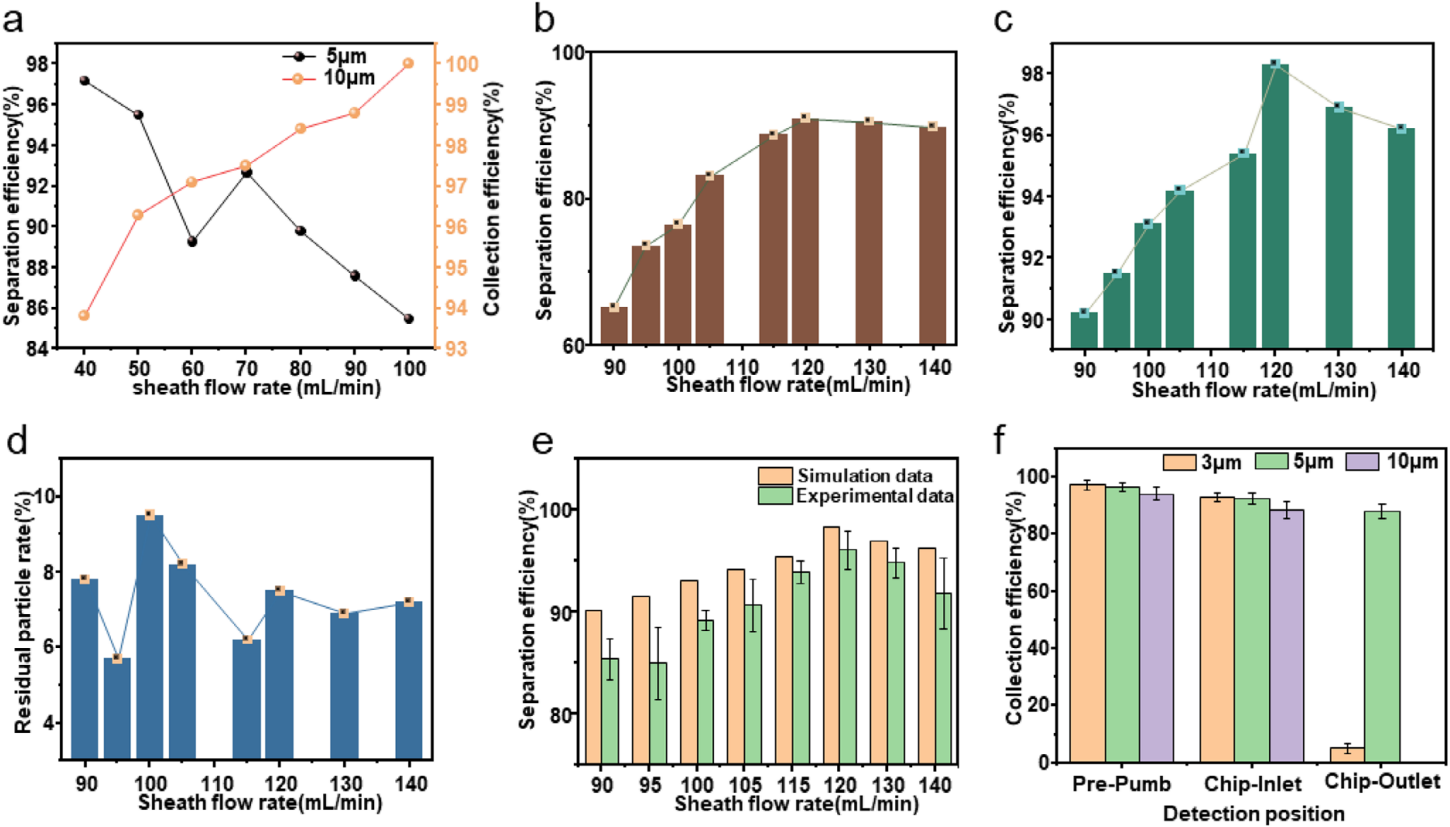
Evaluation results of the microfluidic chip's performance metrics, in both simulations and experiments, 100 particles were used for each particle type. (a) Collection rate of 5µm particles in the next‐stage channel and collection rate of 10µm particles in the collection zone at different flow rates of sheath flow I. (b) Separation efficiency of the chip for 5µm particles at different flow rates of sheath flow II in simulation experiments. (c) Collection efficiency of the chip for 5µm particles at different flow rates of sheath flow II in simulation experiments. (d) Particle retention rate within the microfluidic chip. (e) Comparison between simulation and actual experimental results at different flow rates of sheath flow II. (f) Experimental of the end‐to‐end bioaerosol transport efficiency. Relative collection efficiency of aerosolized particles at three different sizes (3, 5 and 10 µm) measured at key nodes of the sampling path. The “pre‐pump” stage represents the efficiency after passing through the sampling tube, while “chip Inlet” denotes the efficiency after the air pump, just before entering the microfluidic chip. The term “chip‐outlet” denotes the full‐path collection efficiency from the sampling inlet to OutletIof the microfluidic chip (i.e., the electrode detection region).

As indicated by Formulas (1) and (2), when the inlet flow rate is fixed, changing the sheath flow rate alters the width and velocity of the focused particle stream. As the velocity of sheath flow I increases, the separation efficiency for large 10 µm impurity particles gradually improves; however, this also causes more target particles to be retained in the impurity collection zone, leading to a decrease in collection efficiency. When analyzing the performance of the first‐stage sheath flow, we found that the chip's overall performance was optimal at a flow rate of 50 mL/min, at which point the impurity particle separation rate reached 96.28% (Figure 4a). Furthermore, as the velocity of sheath flow II increases, the collection rate of 5 µm particles gradually improves, eventually stabilizing at around 91% (Figure 4b). The particle retention rate in the channel at different flow rates was assessed by counting the collected particles at the outlet (Figure 4c). Additionally, according to the simulation results, the separation performance for 5 µm particles was best when the sheath flow II velocity reached 120 mL/min, with the proportion of 5 µm particles at Outlet I reaching 98.3% (Figure 4d).

In the actual experiments, we used 3 µm, 5 µm, and 10 µm microspheres to validate the chip's separation performance. The experiments for each sheath flow velocity were repeated five times. For microfluidic performance validation, each aerosol generator was operated for 2 min to ensure sufficient particle statistics for separation analysis, and a petri dish coated with vaseline was placed at Outlet I to collect the aerosol particles. When the flow rate of sheath flow I was 50 mL/min and sheath flow II was 120 mL/min, the separation efficiency for 5 µm particles reached 95.6% (Figure 4e). An analysis of the particle counts under a microscope showed that the difference between the experimental and simulation results did not exceed 5%. Several reasons may account for this discrepancy: (1) Bonding errors between the two PDMS substrates could create gaps under fluid pressure; (2) Manual errors can occur during the soft lithography fabrication process; (3) The simulation neglected inter‐particle interactions.

To measure the particle collection efficiency along the entire pathway from the sampling port to the detection region, fluorescent microspheres were utilized as surrogate aerosol particles (Figure 4f). Three sampling points were established, namely upstream of the air pump, before the chip inlet, and at Chip Outlet I (the electrode detection region). Polycarbonate membrane filters with a pore size of 0.4 µm were employed to capture aerosol particles. In this study, 5 µm particles were defined as target viral aerosol particles, and the staged collection efficiency of this particle size was adopted to characterize the overall transmission performance. The experimentally measured collection efficiencies for 5 µm particles were 96.5 ± 1.5% at the pre‐pump position, 92.3 ± 2.0% at the chip inlet, and 88.2 ± 2.4% across the entire system. In comparison, the full‐system recovery rates were only 5.3 ± 1.7% for 3 µm particles and approximately 0% for 10 µm particles, revealing pronounced size‐selective transmission that effectively rejects undesired background particles. Given that the EIS sensing module was directly integrated at the outlet of the microfluidic separation chip, the overall recovery of 5 µm target particles was adopted as the validated end‐to‐end efficiency spanning airborne sampling to electrode‐based detection.

Although the morphology of microspheres is more idealized, the study remains highly valuable as a reference for microbial aerosol research.

The microfluidic chip was fabricated using soft lithography; the structural design was based on the aforementioned simulation optimization results, and a mask was created accordingly. During the fabrication process, we used the SU8‐2100 series of photoresist, forming channels with a height of approximately 100 µm; a mold was obtained after UV exposure and development. Using PDMS as the cover and substrate, the microfluidic chip was produced through processes including vacuum degassing, baking, bonding, and drilling. The selected PDMS material has a degree of biocompatibility and does not alter the biological properties of the samples. By utilizing a microfluidic chip for efficient separation and purification of target particles, combined with EIS signal analysis and machine learning classification, the system demonstrates significant advantages in speed, sensitivity, and automated processing, providing a feasible solution for the development of portable, on‐site detection platforms.

### 3.2 Virus Aerosol Impedance Detection and Classification Results

**FIGURE 5:**
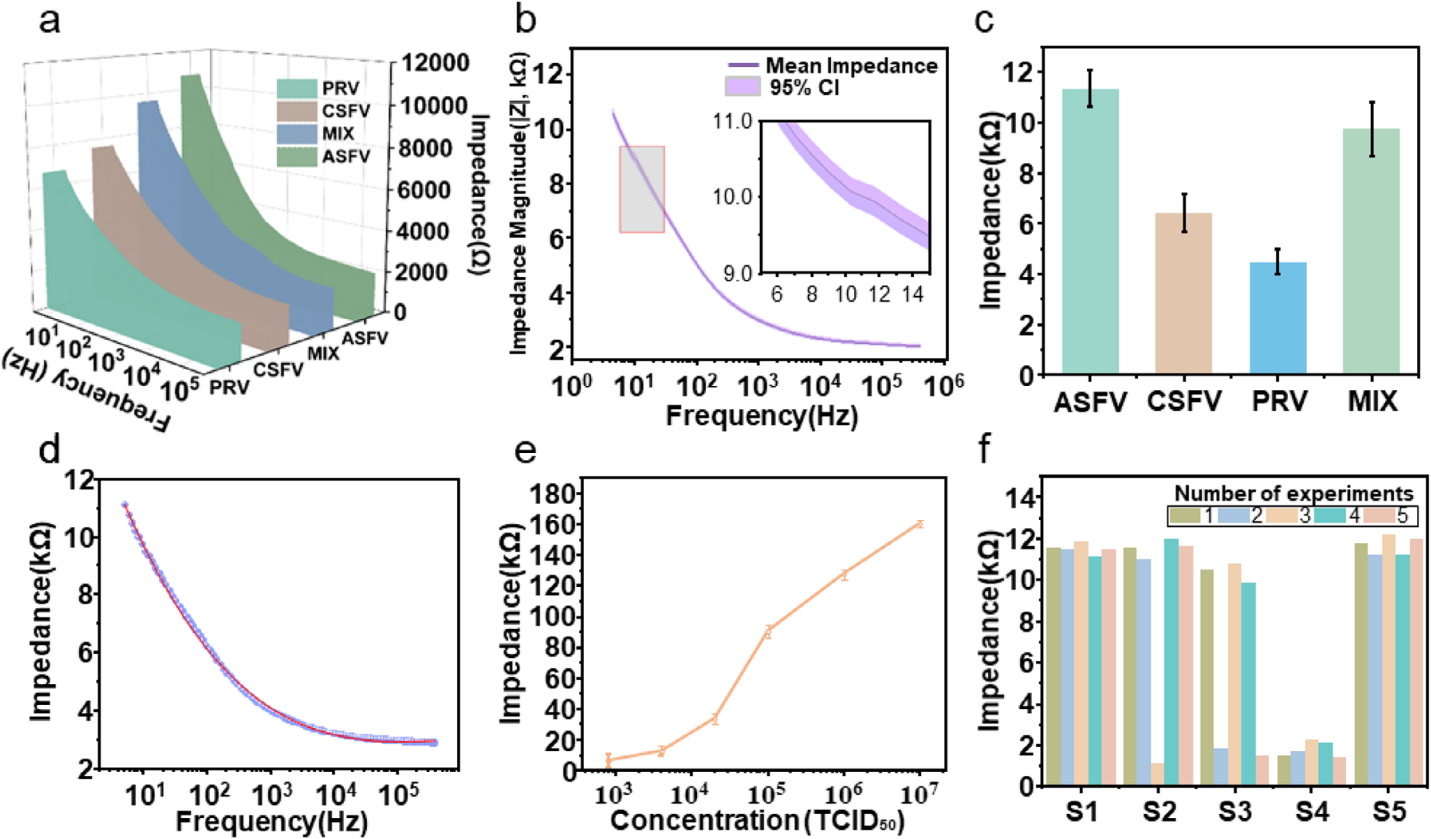
Aerosol impedance spectra and data analysis. (a) Impedance curves in the 5 Hz–1 MHz frequency range. (b) Confidence bands from multiple repeat experiments and a magnified view of the local data near 10 Hz, with results showing that device and sample differentiation is optimal at 10 Hz. (c) Impedance data for ASFV, CSFV, PRV, and mixed virus samples at a detection frequency of 10 Hz. (d) Fitted curve of the detection model for samples containing ASFV. (e) Impedance data at a detection frequency of 10 Hz for ASFV samples after being diluted by factors of 1:10, 1:100, 1:500, 1:2500, and 1:12500. (f) Plot of results from 5 repeated experiments on 5 sets of anomalous data. The plot suggests that the reason for false negatives may be due to the virus being embedded within the aerosol particles.

Based on the processed impedance spectra, the impedance responses of ASFV, CSFV, PRV, and mixed virus aerosol samples were measured over a frequency range of 5 Hz–1 MHz using an impedance analyzer (Figure 5a). Distinct variations in impedance behavior were observed among different virus aerosol samples, consistent with the differences revealed by the equivalent circuit analysis. To assess the reproducibility of the detection results, parallel analyses were performed on multiple sets of sample data, and a 95% confidence interval band was plotted (Figure 5b). In the 5–15 Hz range, the confidence band was narrow, indicating low fluctuation in the results of repeated experiments and good detection stability. In the low‐frequency region (5–15 Hz), impedance differences among ASFV, non‐target viruses, and mixed aerosols were maximized, while the confidence interval across repeated measurements reached its minimum near 10 Hz, indicating superior robustness. Moreover, according to the equivalent circuit analysis, impedance at this frequency is dominated by interfacial charge transfer and adsorption‐related elements, which are most sensitive to virus–antibody interactions. Therefore, 10 Hz was selected as the optimal characteristic frequency for subsequent quantitative analysis and machine learning classification.

Detection frequency for this study, to be used for subsequent concentration gradient analysis and classification model training. The results showed that the impedance differences among the sample types were most significant in the low‐frequency range (5–15 Hz), where the impedance values for ASFV and mixed virus samples were both above 9 kΩ, while those for CSFV and PRV samples were approximately 5–6 kΩ (Figure 5c). At high frequencies, the impedance differences were markedly reduced, approaching the background level of the solution. This result demonstrates the effectiveness of the method. We fitted the impedance data according to the established circuit model (Figure 5d) and calculated that when ASFV aerosol samples were detected, the circuit element parameters were:

(11) $$\begin{array}{ccc} Z_{Virus}\left(\omega \right) & & \approx +2715\frac{1}{7.2\times 10^{-7}\cdot {\left(j\omega \right)}^{0.82}+\frac{1}{3530}}+4850\\ & & \cdot \frac{\tanh\left(\sqrt{0.08j\omega }\right)}{\sqrt{0.08j\omega }} \end{array}$$

After establishing 10 Hz as the characteristic detection frequency, ASFV aerosol samples were tested at different concentration gradients to evaluate the method's sensitivity (Figure 5e). The sample concentrations were set at serial dilutions of 1:10, 1:100, 1:500, 1:2500, and 1:12500 (dilution factor, based on TCID_50_/mL). The impedance values showed a gradual downward trend as the concentration decreased, exhibiting a good correlation with the virus content. A four‐parameter logistic model (4PL) was used to fit the concentration‐impedance data, and the fitting formula is:

(12) $$Z=\frac{165330}{1+{\left(\frac{\log_{10}\left(C\right)}{5.03}\right)}^{-8}}+2312(\Omega )\left(R^{2}=0.972\right)$$

The fitted curve showed a high degree of agreement with the measured data, indicating that the method possesses stable response characteristics over a wide concentration range.

Using the impedance response of a PBS blank sample as a control, the mean impedance value and the corresponding 3σ statistical threshold were calculated, yielding a LOD of 188 TCID_50_/mL for the proposed method. This LOD is derived from the liquid‐phase electrochemical readout within the detection region of the microfluidic chip, where airborne aerosol samples collected by the system are transferred into a localized PBS environment to enable stable impedance measurements. Therefore, the reported LOD represents a system‐level detection limit for air‐sampled aerosols expressed in liquid‐equivalent concentration units, rather than a fully air‐normalized concentration. In the present system, the air–liquid conversion of the detection limit is described by the following system‐level relationship:

(13) $$LOD_{air}=\frac{LOD_{liquid}\times V_{liquid}}{Q_{air}\times t_{sample}\times \eta }$$

where *LOD_liquid_* denotes the liquid‐phase detection limit determined from electrochemical impedance statistics,*V_liquid_* represents the effective liquid volume participating in electrochemical detection, *Q*_*air* _is the air sampling flow rate at the chip inlet, *t*_*sample* _is the air sampling duration, and sampling factor(η  =  88.2%) denotes the end‐to‐end sampling efficiency from sampled air to the electrode detection region.

In addition, the total detection time is less than 1 min, supporting the potential applicability of the proposed platform for future on‐site implementation scenarios.

In the 70 sets of ASFV sample detection experiments conducted in this study, we found that five sets of impedance results appeared to be negative. To confirm this phenomenon, these 5 samples were each re‐tested five times at 10 Hz following the experimental procedure shown in Figure 1a (Figure 5f). The results showed that the third test of the second group and the second test of the third group both appeared negative, meaning false‐negative results occurred. The observed false‐negative responses may be attributed to virus particles embedded within larger aerosol carriers, which can limit effective antibody–antigen interactions at the electrode surface [53, 54, 55]. In such cases, the electrochemical interface may be predominantly governed by the physicochemical properties of the carrier particles rather than the viral surface itself. From an EIS perspective, this situation is expected to reduce the effective interfacial perturbation induced by specific binding events, thereby suppressing the contribution of adsorption‐related impedance elements in the equivalent circuit. As a result, the measured impedance response may resemble that of weak‐binding or virus‐free states. It should be emphasized, however, that the proposed “embedded” versus “surface‐attached” virus aerosol model should be regarded as a plausible mechanistic interpretation rather than a validated conclusion, because the present study does not provide direct imaging or localization evidence for the physical distribution of viruses within aerosol particles. Future validation could be pursued by combining microscopic imaging, particle dispersion or pretreatment comparison, and interface‐sensitive measurements to determine whether virus encapsulation within aerosol carriers is indeed responsible for the observed false‐negative behavior.

**FIGURE 6:**
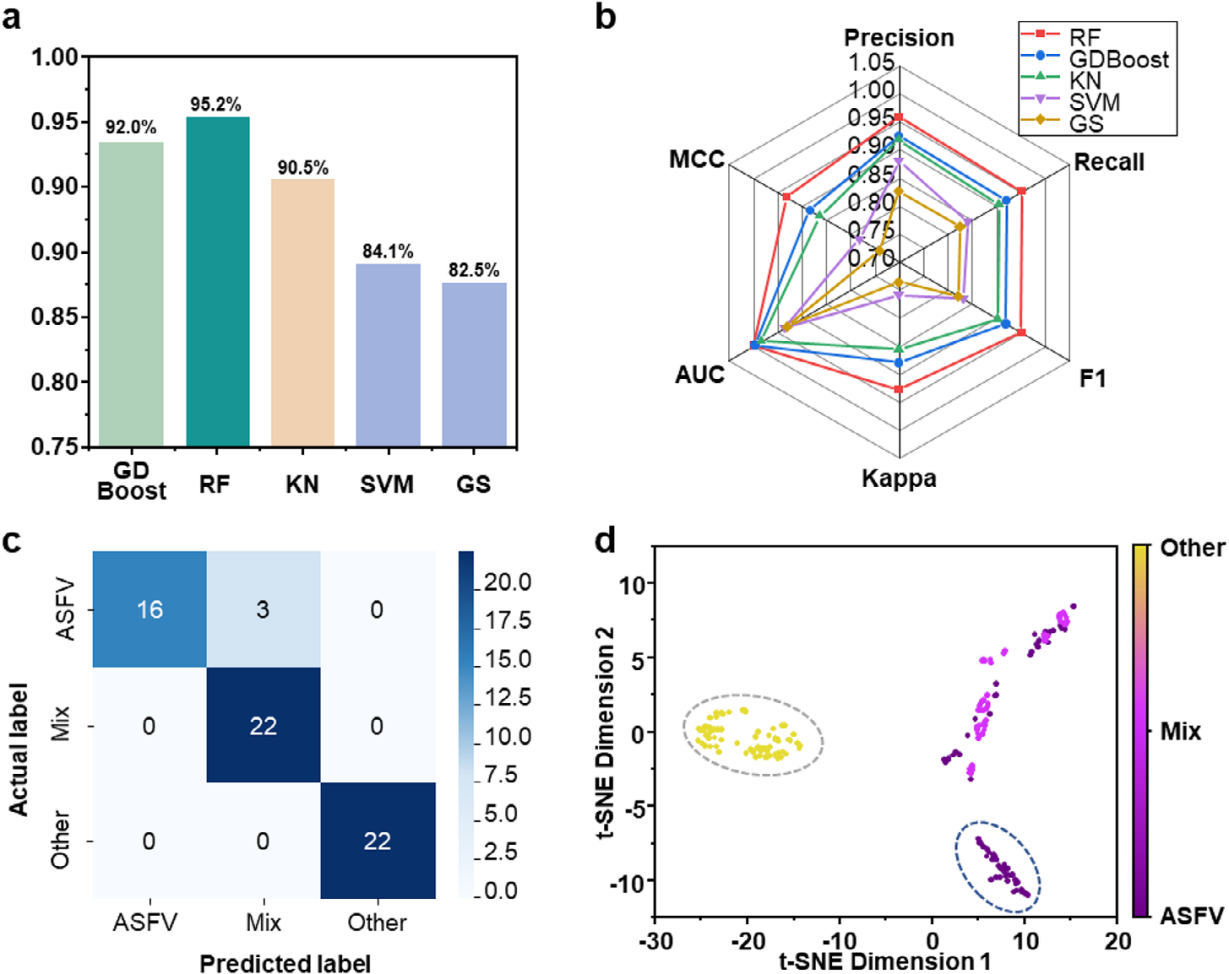
Results of the machine learning classification models. (a) Classification accuracy of the five machine learning models. (b) Radar plot of the evaluation metrics for the five models. (c) Confusion matrix of the classification results from the best‐performing model (RF). (d) Clustering plot by RF for the three class labels (set based on the presence of ASFV and the presence of impurities).

The impedance spectrum information was input into machine learning algorithms for analysis and comparison; the 280 sets of aerosol sample data were randomly divided into a training set and a testing set at a 7:3 ratio. By comparing evaluation metric parameters such as precision, F1‐score, and Kappa, the best‐performing algorithm was found to be RF, with an accuracy of up to 95.2% (Figure 6a, b). The RF model was configured with *n_estimators* = 200, *max_depth* = 10, and *min_samples_split* = 2, using the Gini impurity as the splitting criterion. The ensemble size was chosen to provide sufficient diversity among decision trees, while limiting the maximum tree depth mitigated overfitting under the current dataset scale. The minimum split size and Gini criterion ensured stable node partitioning and computational efficiency during training. Using pure ASFV virus aerosols, mixed virus aerosols, and negative control group aerosols as inputs, the RF classification results showed that 19 sets of ASFV were accurately classified, while the other control groups had almost no misidentifications (Figure 6c). The classification results indicate that the model has a clear boundary for the inter‐class differences between negative and positive samples (Figure 6d).

Notably, the present machine learning framework is designed for screening‐oriented classification based on the presence of the target pathogen and mixed/interfering aerosol conditions, rather than for direct discrimination among all specific virus types. Although ASFV, CSFV, PRV, and mixed aerosols have exhibited distinguishable trends in impedance signal levels at 10 Hz, the current dataset size, labeling strategy, and model architecture are not intended to support a rigorously validated pathogen‐specific multi‐class model. Accordingly, the present results demonstrate that the system enables reliable classification among target‐positive, negative‐control, and mixed aerosol scenarios. Moreover, this work provides a feasible methodological framework for further extension to other specific pathogens.

These results indicate that the detection process is a complex biophysical phenomenon, where the binding of viral aerosol particles to antibodies on the electrode surface induces non‐linear changes in multiple electrochemical parameters such as impedance and phase. From this, we can deduce two points: (1) Our impedance data's decision boundary is non‐linear and exhibits overlap between different categories, suggesting that the inherent characteristics of the aerosol significantly contribute to the circuit model; (2) Feature quantities such as abs and phase are not independent but collectively reflect the state of the virus. Analyzing the classification results, the complex and subtle decision boundaries constructed by the numerous decision trees within the Decision Tree algorithm can automatically capture the intricate non‐linear relationships between features, leading to superior classification performance. Conversely, the SVM and Bayesian algorithms yield less satisfactory classification results when dealing with data exhibiting such overlapping boundaries and non‐independent features (Table 2).

**TABLE 2:** Classification Results Analysis for Each Algorithm.

Algorithm	Compatibility with Data Characteristics	Improvement Direction
**RF**	High. Perfectly aligns with the nonlinear and feature‐correlated nature of EIS data.	Increase n_estimators
**SVM (RBF)**	High. Also capable of handling nonlinear problems, but is highly dependent on data standardization.	Optimize parameters through grid search
**K‐NN**	Medium. Can handle certain nonlinearity, but the decision boundary is coarse and it cannot evaluate feature importance.	Reduce learning rate
**GaussianNB**	Low. Its core assumption of “feature independence” severely contradicts the physical background of the data, leading to model failure.	Feature engineering (e.g., PCA for dimensionality reduction)

Although the RF model demonstrated superior performance in the current study, its applicability may be influenced by dataset scale, aerosol complexity, and target diversity. For larger sample sizes, the ensemble nature of RF is expected to further enhance robustness; however, in highly heterogeneous real‐world aerosols, increased particle diversity and signal overlap may reduce classification margins. In addition, extending the framework to other pathogenic targets may require re‐optimization of feature selection and model parameters. These aspects represent potential limitations and future directions for improving the generalizability of the proposed approach.

The main advantage of the present single‐frequency classification strategy is that it enables dimension‐reduced and computationally efficient classification while retaining the electrochemical information most sensitive to specific binding events on the functionalized electrode surface. In the current system, the electrode was specifically modified for the target analyte, and the selected 10 Hz response captures the most discriminative interfacial information under the present experimental conditions. However, under more complex aerosol backgrounds, the effective binding sites on the electrode surface may become partially blocked or interfered with, which could affect the impedance features at the selected characteristic frequency. In addition, when this framework is extended to other pathogenic aerosol targets, the characteristic frequency may need to be re‐identified according to the corresponding interfacial response of the newly functionalized sensing interface. Therefore, by collecting the spectral information of representative pathogenic microorganisms that may exist in the intended application scenario, the present framework can be flexibly extended to different aerosol‐monitoring contexts through characteristic‐frequency re‐selection and task‐specific model re‐optimization.

**FIGURE 7:**
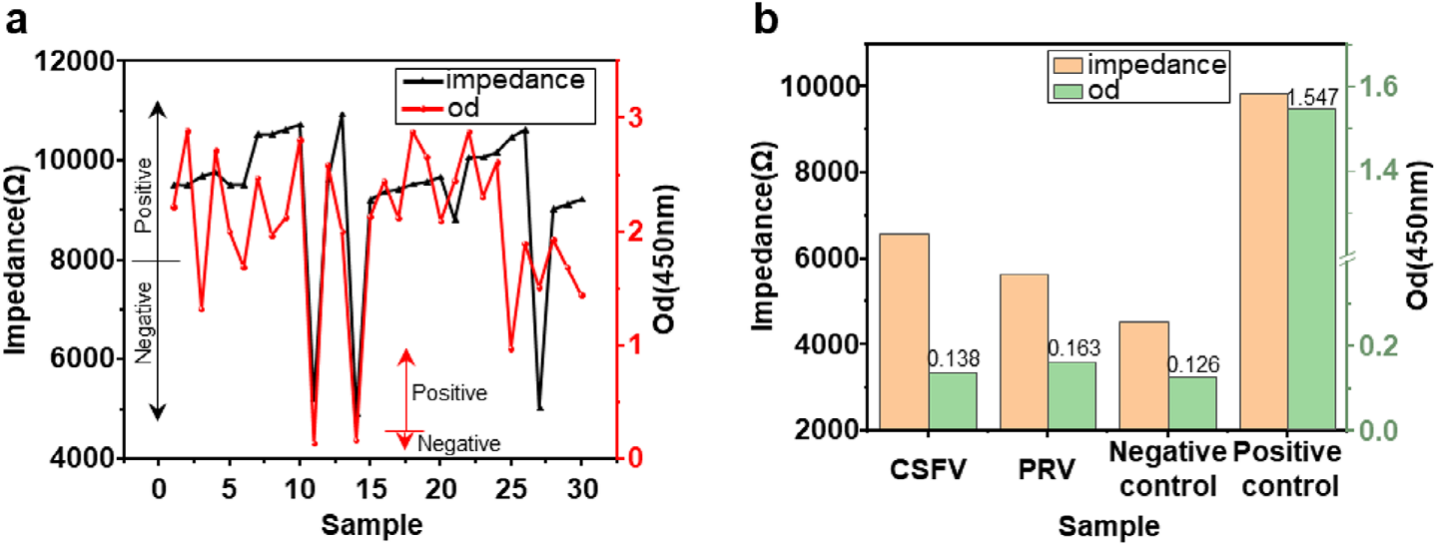
Comparison and validation results with ELISA. (a) Plot of ELISA detection results and the impedance system's detection results for 30 sets of ASFV samples, from which it can be seen that the positive/negative determinations of the two methods are basically consistent. (b) CSFV, PRV, and the negative and positive control samples from the kit were used as the experimental control group.

To further verify the reliability of the system's detection results, CSFV and PRV samples, along with the negative and positive standard liquids provided in the ASFV kit, were selected as a control validation group, and a comparative analysis was performed between the ELISA and the impedance detection results of our system (Figure 7). Additionally, 30 batches of ASFV aerosol samples were randomly selected from the experiments and tested in parallel using both methods. The results showed a high degree of consistency between our system and ELISA in determining positive results; only 1 out of the 30 sample sets had an inconsistent result, corresponding to an agreement rate of 96.7%. Our method for ASFV aerosol detection achieves a complete analysis within one minute.

It should be noted that nucleic‐acid‐based methods such as PCR remain the gold standard in terms of analytical sensitivity and detection accuracy under laboratory conditions. However, these methods typically rely on multi‐step sample processing, including extraction and amplification, as well as laboratory‐grade instrumentation, which limits their practicality for rapid and continuous on‐site monitoring of airborne pathogens. In contrast, the proposed microfluidic–EIS–machine learning platform prioritizes system integration and operational simplicity by enabling automated “sampling–purification–detection–classification” within a single workflow. While this integrated design may sacrifice a degree of analytical precision compared with laboratory‐based assays, it significantly reduces detection time and system complexity, thereby offering clear advantages for on‐site, real‐time screening applications. From an application‐oriented perspective, this trade‐off between analytical sensitivity and deployability is acceptable and, in many scenarios, desirable for early warning and rapid decision‐making in complex environments.

In practical engineering terms, the current platform should be regarded as a laboratory‐scale system that has not yet undergone full field validation. Nevertheless, several features of the present design support its future applicability in aerosol monitoring. First, the workflow integrates aerosol sampling, particle purification, electrochemical sensing, and machine learning‐based analysis, realizing the integration of core functional modules. Second, the system maintains stable discrimination performance under non‐ideal conditions (in the presence of particulate impurities and non‐target interferents), demonstrating a certain tolerance to the complexity of real‐world backgrounds. Third, the characteristic data obtained using a high‐precision impedance analyzer provides a practical foundation for future miniaturization via embedded impedance electronics and simplified signal‐processing hardware. As for electromagnetic shielding of the electrical system, shielding strategies based on functional materials and device packaging can be adopted [56, 57, 58, 59, 60]. Collectively, these attributes support the practical potential of the proposed system for future portable aerosol monitoring applications.

### 3.3 Statistical Analysis

In this study, impedance spectra were preprocessed using a median‐based averaging method to suppress transient fluctuations while preserving intrinsic spectral features. For machine learning analysis, impedance parameters at 10 Hz — including magnitude, phase angle, real part, and imaginary part — were extracted and standardized by z‐score normalization before model training. Unless otherwise stated, all data are expressed as mean ± SD. A total of 280 impedance datasets were employed for machine learning, consisting of 70 samples for each category: ASFV, CSFV, PRV, and mixed aerosols. The dataset was randomly divided into training and test sets at a 7:3 ratio, with five‐fold cross‐validation applied to the training set. The 95% confidence intervals presented in the impedance repeatability analysis were derived from repeated measurements. For particle‐tracking simulations used in chip performance assessment, 100 particles of each size were released under each condition. Statistical interpretations were mainly based on descriptive statistics, confidence interval estimation, and repeatability analysis of replicate measurements. Raw data processing and curve fitting were conducted using Origin software, whereas all machine learning analyses were implemented in Python with standard scientific computing and machine learning libraries.

## 4 Conclusions

This paper proposes an integrated microbial aerosol detection system combining a microfluidic chip with Puri‐focusing functionality, EIS and machine learning. The microfluidic chip was designed and fabricated using soft lithography technology. The chip achieved an actual separation efficiency of 95.8% for 5 µm target microbial particles under dual‐stage sheath flow rates of 50 mL/min and 120 mL/min. We selected ASFV as the representative microorganism and conducted impedance data detection on a SPE. Based on this data, an impedance‐based qualitative and quantitative model was established to probe the interaction state of viral particles within aerosol carriers. In the experiment, the impedance sampling frequency was set to 13.39 k Sa/s, the feature detection frequency was 10 Hz, the detection voltage was 300 mV, and the stable system inlet flow rate was 12.5 mL/min. To validate the system's accuracy, we compared our detection results with the widely commercialized ELISA, achieving a concordance rate of 96.7%. The false‐negative results observed in the experiment might be attributed to viruses being encapsulated within aerosol particles, thereby obstructing effective antibody binding on the electrode surface. These observations support the plausibility of the proposed ‘embedded’ and ‘surface‐attached’ virus aerosol interpretation. To achieve automatic classification of the detection results, five commonly used machine learning algorithms were evaluated in this study. Ultimately, the RF algorithm was selected as the classification model, achieving a classification accuracy up to 95.2%.

These results demonstrate that the proposed integrated microfluidic–EIS–machine learning platform provides a feasible and sensitive approach for the detection of airborne microorganisms, as validated under controlled laboratory conditions. These observations further suggest that the physical state of viruses within aerosol carriers, particularly the distinction between embedded and surface‐attached configurations, may influence detection performance, underscoring a potential limitation of the current approach and the need for further mechanistic investigations. By enabling real‐time impedance monitoring with a specialized biosensor, the proposed system facilitates rapid screening of target microbial aerosol particles and provides a technical foundation for future development toward automated and on‐site epidemic surveillance and control. Furthermore, this integrated strategy may be extended to the monitoring of other aerosol‐transmitted pathogenic microorganisms and their advanced biomedical applications [61, 62, 63].

National Key Research and Development Program for Young Scientists (2022YFD2000200). Key Research and Development Program (Sub‐project) of Jiangsu Province (BE2022052‐2; BE2023017‐2). National Natural Science Foundation of China (General Program) (32572198).

## Conflicts of Interest

The authors declare no conflicts of interest.

## Supporting information




**Supporting File**: advs75184‐sup‐0001‐SuppMat.docx.

## Data Availability

The data that support the findings of this study are available from the corresponding author upon reasonable request.
