# Supplementary material for: Machine‐Learning Microfluidic Minute‐Scale Microorganism Metrics Monitoring(M6)
Source: Adv Sci (Weinh). 2026 Apr 15:e21106. Online ahead of print. doi: 10.1002/advs.202521106 (PMC13334659; doi:10.1002/advs.202521106)
Supplement: Supplementary file 1 — Supporting File: advs75184‐sup‐0001‐SuppMat.docx. [file ADVS-9999-e21106-s001.docx]

**MACHINE-LEARNING MICROFLUIDIC MINUTE-SCALE MICROORGANISM METRICS MONITORING(M6)**

Ning Yang^1,2#^, Jiahao Ding^1,#^, Si Chen^2,5,#^, Lijie Yan^2,6,7#^, Shichao Ding^2,3,#^, Lavonda Li^2,4,#^, Junyi Sun^2,6^, Haodong Liu^1^, Tongge Li^1^, Ning Liu^1^, Mingji Wei^1^, Xiaoyong Zhu^1^, Xiaobo Zou^8^, Shouqi Yuan^3^, Xingcai Zhang^2,3,4*^

^1^School of Electrical and Information Engineering, Jiangsu University, Zhenjiang, China, 212013.

^2^ World Tea Organization, Cambridge, MA, USA, 02139.

^3^ School of Engineering, University of California, San Diego, La Jolla, CA, USA, 92093.

^4^ Department of Materials Science and Engineering, Stanford University, Stanford, CA, USA, 94305.

^5^ Fluid Machinery Center, Jiangsu University, Zhenjiang, China, 212013.

^6^ School of Medicine, Stanford University, Stanford, CA, USA, 94305.

^7^ Fuwai Central China Cardiovascular Hospital, Central China Fuwai Hospital of Zhengzhou University, Zhengzhou, China, 450003.

^8^School of Food and Biological Engineering, Jiangsu University, Zhenjiang, China, 212013.

*Corresponding email: [xiz292@ucsd.edu](about:blank), drtea1@wteao.com.

**Support Imformation:**

**Table S1. Analysis of Several Classification Algorithms**

**Figure S1. Experiment on IDEs**

**Table S2. Analysis of Figure S1 Results**

**Figure S2. Design Dimensions and Physical Image of Microfluidic Chip**

**S1. Median-based Impedance Spectrum Processing(pseudocode)**

**Table S1. Analysis of Several Classification Algorithms**

The advantages and disadvantages of classification learning algorithms are discussed here. The algorithm objects under discussion are as follows:

1. ***Regularization Algorithm*：**Techniques used to reduce the risk of overfitting in machine learning

models.

| **Branch Algorithm** | **Advantages** | **Disadvantages** |
| --- | --- | --- |
| **L1 Regularization (Lasso Regularization)** | 1. Can be used for feature selection by driving the coefficients of unimportant features to zero.  2. Can solve the problem of multicollinearity. | 1. May select fewer features for high-dimensional data.  2. Requires adjustment of the regularization parameter. |
| **L2 Regularization (Ridge Regularization)** | 1. Can solve the problem of multicollinearity.  2. Insensitive to outliers. | 1. Not suitable for feature selection, as all features are taken into account.  2. Requires adjustment of the parameter. |
| **Elastic Net Regularization** | 1. Combines the advantages of L1 and L2 regularization, capable of addressing both multicollinearity and feature selection.  2. Allows adjustment of two regularization parameters to balance the effects of L1 and L2 regularization. | Requires adjustment of two regularization parameters. |
| **Dropout Regularization** | 1. Reduces overfitting in neural networks by randomly deactivating neurons during training.  2. No additional parameter adjustment is needed. | 1. During inference, the deactivated neurons need to be considered, increasing computational cost.  2. May require more training iterations. |
| **Bayesian Ridge and Lasso Regression** | 1. Incorporates Bayesian thinking and can provide uncertainty estimation for parameters.  2. Can automatically determine the regularization parameter. | 1. High computational cost, especially for large datasets.  2. Not suitable for all types of problems. |
| **Early Stopping** | 1. Reduces overfitting in neural networks by monitoring performance on the validation set.  2. Simple and easy to use, with no need for additional parameter adjustment. | Requires careful selection of the training stopping point; stopping too early may lead to underfitting. |
| **Data Augmentation** | 1. Reduces the risk of model overfitting by increasing the diversity of training data.  2. Suitable for fields such as image classification. | Increases the cost of generating and managing training data. |

1. ***Ensemble Algorithms*：**Techniques that combine multiple weak learners (usually base models) into

a strong learner.

| **Branch Algorithm** | **Advantages** | **Disadvantages** |
| --- | --- | --- |
| **Bagging (Bootstrap Aggregating)** | 1. Reduces model variance and the risk of overfitting.  2. Supports parallel processing, suitable for large-scale data. | 1. Not suitable for handling highly skewed class distributions.  2. Difficult to interpret the prediction results of the combined model. |
| **Random Forest** | 1. Based on Bagging, it reduces variance.  2. Capable of handling high-dimensional data and large-scale features.  3. Provides feature importance evaluation. | 1. Difficult to adjust a large number of hyperparameters.  2. Sensitive to noise and outliers. |
| **Boosting** | 1. Improves model accuracy.  2. Can automatically adjust the weights of weak learners.  3. Suitable for imbalanced class distributions. | 1. Sensitive to noisy data.  2. Training time may be long. |
| **AdaBoost (Adaptive Boosting)** | Capable of handling high-dimensional data and large-scale features, with low sensitivity to outliers. | Sensitive to noise and outliers. |
| **Gradient Boosting** | Provides high prediction performance and is relatively stable against noise and outliers. | Requires adjustment of multiple hyperparameters. |
| **XGBoost, LightGBM (Extreme Gradient Boosting and Light Gradient Boosting Machine)** | Both are variants of the gradient boosting algorithm, featuring high efficiency and scalability. | - |
| **Stacking** | 1. Can combine multiple models of different types.  2. Provides higher prediction performance. | 1. Requires more computing resources and data.  2. Higher complexity, making hyperparameter adjustment more difficult. |
| **Voting** | 1. Simple and easy to use, with straightforward implementation.  2. Can combine multiple models of different types. | 1. Has high performance requirements for weak learners.  2. Does not consider the weights of individual models. |
| **Deep Learning Ensemble** | 1. Can leverage the powerful representation ability of neural network models.  2. Offers various ensemble methods, such as voting and stacking. | 1. Long training time and requires a large amount of computing resources.  2. Hyperparameter adjustment is more complex. |

1. ***Decision Tree Algorithms*：**Supervised algorithms based on a tree-like structure, used for

classification and regression tasks. They build a tree structure through a series of splits, where each internal node represents a feature test and each leaf node represents a class or numerical output.

| **Branch Algorithm** | **Advantages** | **Disadvantages** |
| --- | --- | --- |
| **ID3 - Iterative Dichotomiser 3** | 1. Simple and easy to understand, with easily interpretable generated trees.  2. Capable of handling classification tasks. | 1. Limited handling of numerical attributes and missing values.  2. Prone to overfitting; the generated tree may be very deep. |
| **C4.5** | 1. Can handle both classification and regression tasks.  2. Capable of handling numerical attributes and missing values.  3. Uses information gain for feature selection when generating trees, making it more robust. | 1. Sensitive to noise and outliers.  2. The generated tree may be overly complex and requires pruning to reduce the risk of overfitting. |
| **CART (Classification and Regression Trees)** | 1. Can handle both classification and regression tasks.  2. Provides good support for numerical attributes and missing values.  3. Uses Gini impurity or mean squared error for feature selection, making it more flexible. | The generated tree may be deep and requires pruning to avoid overfitting. |
| **Random Forest** | 1. Based on decision trees, it reduces the overfitting risk of decision trees.  2. Capable of handling high-dimensional data and large-scale features.  3. Provides feature importance evaluation. | 1. Difficult to adjust a large number of hyperparameters.  2. Sensitive to noise and outliers. |
| **Gradient Boosting Trees** | 1. Provides high prediction performance and is relatively stable against noise and outliers.  2. Suitable for both regression and classification tasks.  3. Can use different loss functions. | 1. Requires adjustment of multiple hyperparameters.  2. Training time may be long. |
| **Multi - output Trees** | 1. Capable of handling multi - output (multi - target) problems.  2. Can predict multiple related target variables. | Requires a large amount of data to train effective multi - output trees. |

1. ***Support Vector Machines (SVM)*：**Powerful supervised learning algorithms used for classification

and regression tasks. They find the optimal hyperplane to separate data into different classes or fit a regression function.

| **Branch Algorithm** | **Advantages** | **Disadvantages** |
| --- | --- | --- |
| **Linear Support Vector Machine** | 1. Effective in high - dimensional spaces, suitable for high - dimensional data.  2. Can be extended to non - linear problems by selecting different kernel functions.  3. Has strong generalization ability. | 1. Sensitive to large - scale datasets and the number of features.  2. Sensitive to noise and outliers. |
| **Non - linear Support Vector Machine** | 1. Can handle non - linear problems.  2. Can adapt to different types of data by selecting appropriate kernel functions. | 1. For complex non - linear relationships, appropriate kernel functions and parameters may need to be selected.  2. High computational complexity, especially for large datasets. |
| **Multi - class Support Vector Machine** | Can handle multi - class classification problems. Common methods include the One - vs - One and One - vs - Rest strategies. | 1. In the One - vs - One strategy, multiple classifiers need to be constructed.  2. In the One - vs - Rest strategy, class imbalance problems may occur. |
| **Kernel - based Support Vector Machine** | 1. Can handle non - linear problems.  2. Usually uses the Radial Basis Function (RBF) as the kernel function.  3. Suitable for complex data distributions. | 1. Needs to select appropriate kernel functions and related parameters.  2. May have overfitting risks for high - dimensional data. |
| **Sparse Support Vector Machine** | 1. Introduces sparsity, where only a few support vectors contribute to the model.  2. Can improve the training and inference speed of the model. | Not suitable for all types of data; its performance may be poor for certain data distributions. |
| **Bayesian Kernel Support Vector Machine** | 1. Combines kernel methods and Bayesian methods, with probabilistic inference capabilities.  2. Suitable for small - sample and high - dimensional data. | High computational complexity, may not be applicable for large - scale datasets. |
| **Imbalanced Class Support Vector Machine** | 1. Specifically designed to handle class imbalance problems.  2. Balances the impact of different classes by adjusting class weights. | 1. Needs to adjust weight parameters.  2. For extremely imbalanced datasets, other methods may be required. |

1. ***Clustering Algorithms*：**Unsupervised learning algorithms used to group data into clusters or groups

with similarity.

| **Branch Algorithm** | **Advantages** | **Disadvantages** |
| --- | --- | --- |
| **K - means Clustering** | 1. Simple and easy to understand, easy to implement.  2. Suitable for large - scale data.  3. Fast speed, applicable for many applications. | 1. Needs to pre - specify the number of clusters K.  2. Sensitive to the selection of initial cluster centers.  3. Sensitive to outliers and noise.  4. Suitable for convex clusters. |
| **Hierarchical Clustering** | 1. Does not require pre - specifying the number of clusters.  2. Can generate a hierarchical cluster structure.  3. Suitable for clusters of irregular shapes. | 1. High computational complexity, not suitable for large - scale data.  2. Poor interpretability of results. |
| **Density - based Clustering** | 1. Can discover clusters of arbitrary shapes.  2. Relatively robust to noise and outliers.  3. Does not require pre - specifying the number of clusters. | 1. Sensitive to parameter selection.  2. Not suitable for cases where data density varies greatly. |
| **Spectral Clustering** | 1. Can discover clusters of arbitrary shapes.  2. Suitable for clusters of irregular shapes.  3. Not affected by the selection of initial cluster centers. | 1. High computational complexity, not applicable for large - scale data.  2. Needs to carefully select the similarity matrix and the number of clusters. |
| **DBSCAN** | 1. Can automatically discover clusters of arbitrary shapes.  2. Relatively robust to noise and outliers.  3. Does not require pre - specifying the number of clusters. | 1. For high - dimensional data, special attention must be paid to parameter selection.  2. May perform poorly when data density varies significantly. |
| **EM Clustering** | 1. Suitable for mixture models and can discover probability distribution clusters.  2. Suitable for cases where data has missing values. | 1. Sensitive to the selection of initial parameters.  2. For high - dimensional data, special attention must be paid to parameter selection. |
| **Fuzzy Clustering** | 1. Can assign each data point to multiple clusters, considering the uncertainty of data.  2. Suitable for fuzzy classification problems. | 1. High computational complexity.  2. Poor interpretability of results. |

1. ***Bayesian Algorithms*：**A statistical method based on Bayes' theorem, used to handle uncertainty

and probabilistic inference.

| **Branch Algorithm** | **Advantages** | **Disadvantages** |
| --- | --- | --- |
| **Naive Bayes** | 1. Simple, easy to understand and implement.  2. Performs well on small - scale data and high - dimensional data.  3. Can be used for tasks such as classification and text classification. | 1. Based on a strong feature independence assumption, which may not be suitable for data with complex correlations.  2. Sensitive to imbalanced data and noisy data. |
| **Bayesian Network** | 1. Can represent and infer complex probabilistic relationships and dependencies.  2. Supports handling incomplete data and missing values.  3. Suitable for domain modeling and decision support systems. | 1. Learning the model structure and estimating parameters can be complex.  2. For large - scale data and high - dimensional data, the computational cost may be high. |
| **Gaussian Process** | 1. Can model non - linear relationships and uncertainty.  2. Provides confidence interval estimation.  3. Suitable for regression and classification tasks. | 1. High computational complexity, not suitable for large - scale data.  2. Needs to select appropriate kernel functions and hyperparameters. |
| **Bayesian Optimization** | 1. Used for optimizing black - box functions, such as hyperparameter tuning.  2. Can find the optimal solution in a small number of iterations.  3. Suitable for complex and expensive optimization problems. | 1. Relatively high computational cost.  2. Needs to carefully select priors and sampling strategies. |
| **Variational Bayesian** | 1. Used for parameter estimation and inference in probabilistic models.  2. Can be used to handle large - scale datasets.  3. Provides a framework for approximate inference. | 1. Approximate inference may introduce estimation errors.  2. Careful model selection and parameter selection are required. |
| **Bayesian Deep Learning** | 1. Combines deep learning and Bayesian methods, providing uncertainty estimation.  2. Suitable for small - sample learning and model uncertainty modeling. | 1. High computational complexity and long training time.  2. Complex hyperparameter adjustment. |

1. ***Artificial Neural Networks*：**Machine learning models designed inspired by the structure of the

human brain, used to handle various tasks, including classification, regression, image processing, and natural language processing.

| **Branch Algorithm** | **Advantages** | **Disadvantages** |
| --- | --- | --- |
| Feedforward Neural Networks (FNNs) | 1. Suitable for various tasks, including classification and regression.  2. Has strong representation ability and can capture complex non - linear relationships.  3. Provides a foundation for deep learning problems. | 1. Prone to overfitting for small - sample data.  2. Requires a large amount of labeled data for training. |
| Convolutional Neural Networks (CNNs) | 1. Specifically used for image processing and computer vision tasks.  2. Effectively captures local features in images through convolutional layers.  3. Has translation invariance. | 1. Requires a large amount of labeled image data for training.  2. May perform worse than feedforward neural networks in tasks in other fields. |
| Recurrent Neural Networks (RNNs) | 1. Suitable for sequence data, such as natural language processing and time series analysis.  2. Has recurrent connections and can handle sequence data of variable length.  3. Has memory ability and can capture temporal dependencies. | 1. Suffers from the vanishing gradient problem, leading to performance degradation for long sequences.  2. High computational complexity, not suitable for large - scale data and deep networks. |
| Long Short - Term Memory (LSTM) | 1. Solves the vanishing gradient problem of RNNs.  2. Suitable for modeling long sequences.  3. Has achieved significant success in fields such as natural language processing. | 1. High computational complexity.  2. Requires a large amount of data to train deep LSTM networks. |
| Gated Recurrent Unit (GRU) | 1. Similar to LSTM but with fewer parameters and lower computational complexity.  2. Performs comparably to LSTM in some tasks. | 1. May perform worse than LSTM in some complex tasks. |
| Self - attention Model (Transformer) | 1. Suitable for tasks such as natural language processing and sequence modeling.  2. Can be parallelized, with high computational efficiency.  3. Performs well on large - scale data and deep models. | 1. Requires a large amount of data for training.  2. A relatively new model, may not be suitable for all tasks. |
| Generative Adversarial Networks (GANs) | 1. Used for data and image generation, as well as unsupervised learning.  2. Generates high - quality samples.  3. Has achieved significant success in fields such as image generation and style transfer. | 1. High training complexity and poor stability, requiring careful adjustment of hyperparameters.  2. May suffer from mode collapse in some task |

**Figure S1. Experiment on IDEs**

Regarding electrode selection, we also conducted experiments using interdigitated electrodes (IDEs). However, the experimental results indicated that the detection performance of the IDEs was comparatively unstable, leading us to ultimately select the Screen-Printed Electrode (SPE) as the working electrode.


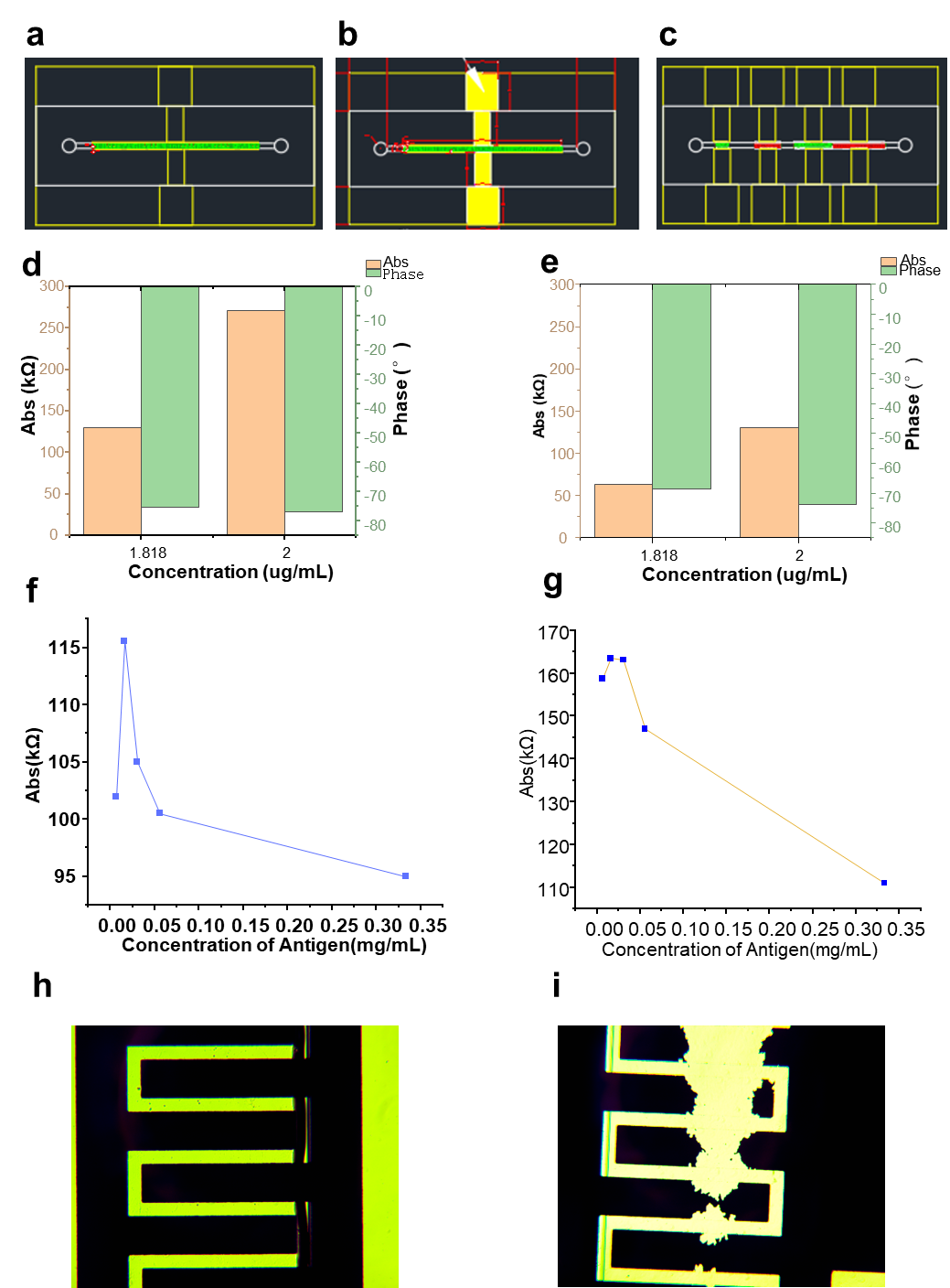


**Fig. S1** (a~c) ​​Electrode Gap Size:a:100-50μm, b:50-50μm, c:50-50; (d~e) In the experiment where the antigen served as the electrode substrate and the antibody was the detection target, d represents the detection result from Electrode a, and e represents the detection result from Electrode b. The impedance values between the two show a significant difference, with potential reasons detailed in Table S2. (f~g) f represents the results of continuous detection without a PBS rinse, while g represents the results of detection after rinsing. (h~i)Regarding our cleaning experiments, these results are from ultrasonic cleaning: h represents the normal/initial state, and i represents the result after cleaning. The findings indicate that the gold layer on the interdigitated electrodes (IDEs) is relatively fragile, and conversely, failing to perform cleaning would significantly impact the experimental results.

**Table S2. Analysis of Figure S1 Results**

Analysis of the Reasons for the Discrepancies between the Two Interdigitated Electrode Experiments and Expectations.

| **Possible Cause** | **Core Mechanism** | **Manifestation** |
| --- | --- | --- |
| Virus Charging Effect (Primary) | Charged viruses enrich counterions, forming conductive channels that promote charge transfer. | Rct (Charge Transfer Resistance) decreases. |
| Interfacial Capacitance Change | Viruses alter the interfacial dielectric environment, leading to increased capacitance and decreased capacitive impedance. | Overall impedance decreases at specific frequencies. |
| Altered Probe Interaction | Viruses modify surface charge, affecting the diffusion and proximity of charged probes. | Rct decreases. |
| Molecular Conformational/Displacement Effect | Antibody conformational changes or displacement of nonspecifically adsorbed substances expose active sites. | Rct decreases. |

**Figure. S2. Design Dimensions and Physical Image of Microfluidic Chip**


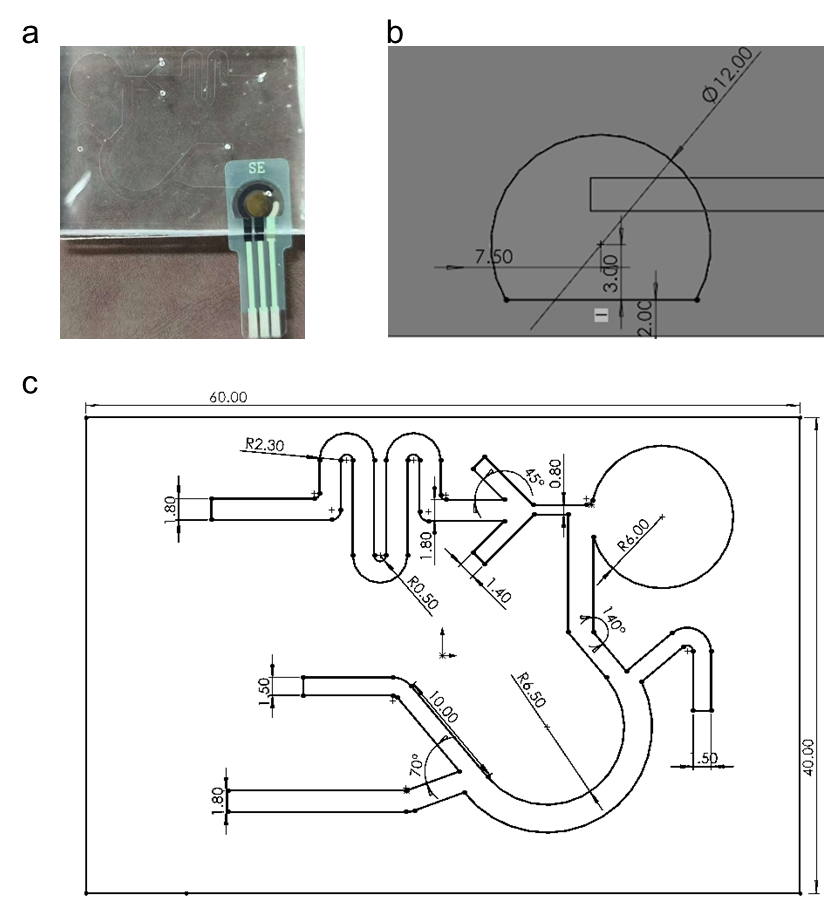


**Fig. S1**. A photograph of the two-layer microfluidic chip fabricated from a PDMS substrate, with detailed dimensions of its constituent structures.

**S1. Median-based Impedance Spectrum Processing(pseudocode)**

Input:

Frequency set:

F = {f1, f2, ..., fN_freq}, covering 5 Hz to 1 MHz

For each sample s (ASFV, CSFV, PRV, and mixed samples):

Repeated impedance measurements acquired by the impedance analyzer:

ABS_rep[s] shape = (N_rep, N_freq) // impedance magnitude |Z|

PHASE_rep[s] shape = (N_rep, N_freq) // phase angle

RE_rep[s] shape = (N_rep, N_freq) // real part Re(Z)

IM_rep[s] shape = (N_rep, N_freq) // imaginary part Im(Z)

/* where N_rep denotes the number of repeated measurements performed at each frequency point, and N_freq denotes the number of frequency points in the measured spectrum (5 Hz–1 MHz). */

Output:

For each sample s:

ABS_med[s] shape = (N_freq,) // median-processed |Z| spectrum

PHASE_med[s] shape = (N_freq,) // median-processed phase spectrum

RE_med[s] shape = (N_freq,) // median-processed Re(Z) spectrum

IM_med[s] shape = (N_freq,) // median-processed Im(Z) spectrum

**Algorithm:**

For each sample s:

For each frequency index j = 1 to N_freq:

// Median aggregation across repeated measurements at the same frequency point

ABS_med[s][j] = median (ABS_rep[s][1..N_rep, j] ) // 50th percentile

PHASE_med[s][j] = median (PHASE_rep[s][1..N_rep, j] ) // 50th percentile

RE_med[s][j] = median (RE_rep[s][1..N_rep, j] ) // 50th percentile

IM_med[s][j] = median (IM_rep[s][1..N_rep, j] ) // 50th percentile

**Notes:**

1) Purpose: This median-based processing is used to obtain robust impedance spectra for impedance curve analysis and characteristic-frequency selection (e.g., identifying 10 Hz as the characteristic frequency).

2) Axis of application: The median operation is applied across repeated measurements (N_rep) at each frequency point, rather than across frequencies.

3) Windowing strategy: No sliding window is used; processing is performed independently at each frequency point.

4) Outlier handling: Outliers are not explicitly removed; extreme values are inherently suppressed by the median operator.

6) Consistency: The same processing is applied consistently to all samples to ensure comparability.
